# Factors contributing to motivation of volunteer community health workers in Ethiopia: the case of four woredas (districts) in Oromia and Tigray regions

**DOI:** 10.1186/s12960-018-0319-3

**Published:** 2018-11-08

**Authors:** Habtamu Abdissa Jigssa, Binyam Fekadu Desta, Hibret Alemu Tilahun, Jen McCutcheon, Peter Berman

**Affiliations:** 1 International Division, John Snow Research and Training Institute, Inc., Addis Ababa, Ethiopia; 2 Department of Global Health and Population, Harvard T.H. Chan School of Public Health, Boston, MA United States of America; 3 John Snow Research and Training Institute, Inc., Fenot Project, Bole Sub City, Woreda 03/05, House No. 2347, PO Box 1988, 1250 Addis Ababa, Ethiopia

**Keywords:** Community health workers, Health Development Army, One-to-five network leaders, Motivation, Volunteer, Ethiopia

## Abstract

**Background:**

The use of community health workers (CHWs) has been considered as one of the strategies to address the growing shortage of health workers, predominantly in low-income countries. They are playing a pivotal role in lessening health disparities through improving health outcomes for underserved populations. Yet, little is known about what factors motivate and drive them to continue working as CHWs. In this study, we aimed to examine factors contributing to the motivation of volunteer CHWs (vCHWs) in Ethiopia currently known as one-to-five network leaders (1to5NLs) and explore variations between attributes of social and work-related determinants.

**Method:**

We conducted a cross-sectional study in four selected woredas (the second lowest administrative structure in Ethiopia, and similar to a district) of Oromia and Tigray regions and interviewed 786 1to5NLs. The effects of each motivational factor were explored using percentage of respondents who agreed and strongly agreed to each of them and Mann-Whitney *U* test.

**Results:**

Individual, community, and health system factors contributed to the motivation of 1to5NLs in this study. Intrinsic desire to have a good status in the community as a result of their volunteer service (81.86%) followed by a commitment to serve the community (81.61%) and to gain satisfaction by accomplishing something worthwhile to the community (81.61%) were some of the factors motivating 1to5NLs in our study. Despite these motivational items, factors such as lack of career development (51.47%), unclear health development army guideline (59.26%), limited supervision and support (62.32%), and lack of recognition and appreciation of accomplishments (63.22%) were the factors negatively affecting motivation of 1to5NLs. Lack of career development, limited supervision and support, and lack of recognition and appreciation of accomplishments were significantly varied between attributes of educational level, marital status, service year as 1to5NLs, and previous volunteer engagement (at *P <* 0.05*).*

**Conclusion:**

Findings of our study indicated that non-financial incentives such as the creation of career development models is the key to motivating and retaining CHWs where they are not receiving stipends. Sustainability of CHW program should consider exploring enhanced innovations to strengthen supportive supervision, development of better mechanisms to publicize the role of CHWs, and improvement of recognition and appreciation schemes for CHWs’ efforts and accomplishments.

## Background

The shortage of well-trained health workers is a challenge to health systems around the world, particularly in low- and middle-income countries. The influx of health workers into high-income countries, increasing morbidity and mortality, inadequately funded and poorly managed and performing health systems, and workload due to various pandemics have made the problem much worse in low-income countries [1, 2].One method of helping to address this multifaceted crisis is by shifting certain tasks to community members with lower qualifications to provide basic health services to their community [2, 3].

For the purposes of this paper, we followed the definition of community health workers (CHWs) proposed by a WHO study group [4]: “CHWs should be members of the communities where they work, should be selected by the communities, should be answerable to the communities for their activities, should be supported by the health system but not necessarily a part of its organization, and have shorter training than professional workers.” Community health workers deliver a wide range of basic services to communities including nutrition; maternal and child health; primary health care; malaria, tuberculosis, and HIV/AIDS prevention and control; mental health; and non-communicable diseases [5]. These workers have been shown to be effective in increasing immunization coverage, improving breastfeeding rates, reducing infant mortality and morbidity, and improving tuberculosis treatment [5–7]. CHWs have also contributed to the prevention and management of communicable and non-communicable diseases and have improved maternal and child health outcomes [3, 6, 7].

While interest in task shifting and using CHWs is high due to its effectiveness and the crises in the health workforce [3–6], the sustainability of such programs is threatened by high rates of attrition [5], particularly among volunteer CHWs (vCHWs) [2]. Frequent turnover of CHWs causes lack of continuity in the relationships established among CHWs, the community, and the health system [2] and increases the cost of CHW programs through repeated identification, screening, selection, and training of CHWs [2]. Therefore, retention of CHWs can be a cost-effective way of improving health service delivery and continuum of care to the community. One mechanism of retention of CHWs is regular assessment and improvement of CHW motivational levels, as retention and performance can be impacted by job satisfaction derived from certain intrinsic and extrinsic motivators [8, 9].

In developing countries, studies on motivation are rarely conducted and are predominantly qualitative [7, 10–12]. Furthermore, assessments on factors contributing to motivation of CHWs are very limited and should be different from usual health staffs particularly due to the fact that they are often not salaried, are workers without prior training on community health, and constitute the outreach workforce directly linking the community with the formal healthcare [13]. Therefore, in this paper, we assessed the factors that may affect motivational level of Health Development Army (HDA) network leaders or one-to-five network leaders (1to5NLs) in rural Ethiopia by doing some assessment using a structured data collection instrument. Selected determinants, such as educational level, marital status, number of years served as a 1to5NL, and previous engagement on some other volunteer service, were also examined to see their effect on the motivational level.

### HDA structure: an overview

Ethiopia has been benefiting from CHWs since 1974. Various names and scopes of practice were given to these volunteers. The current CHW structure, HDA, was introduced in 2011 to mobilize families in order to ensure wider community participation and facilitate community ownership [14, 15].

The approach behind HDA is to create networks of five households/women, led by one woman that practices healthy behavior and gradually influences the rest of the women to acquire skills and attitudinal changes towards healthy behavior. That is, one 1to5NL is responsible for five households in her neighborhood. In some cases, more than one woman may be represented from a single household if they fulfill the criteria. The 1to5NLs report to and are technically supported and supervised by the health extension workers (HEWs), who facilitate and follow up regularly with conversations within the community [11, 16].

Unlike the HEW who is a trained health worker and salaried government employee, the 1to5NL is a female, volunteer, and non-salaried health worker selected by her community. The leader has to be a model family and is selected based on her communication skills, active participation in community development activities, and readiness to teach others. The selection of 1to5NLs is done by HEWs and kebele^1^ council members after a sensitization workshop organized at the gote/village level to identify model households or women who can serve as leaders. The 1to5NLs attend a 15-day (4 h per day) training on health extension packages, led by health center staffs and the HEWs. There are five neighboring women/households under each leader, each of whom receives a 2-day basic training on the health extension packages from their leader. After completion of the training and implementing the packages, the women graduate and are labeled as model families. After graduation, the leader will continue to support the model families in collaboration with the HEWs so that the families continue implementing the packages without any difficulty [11]. The role of the 1to5NLs largely focuses on preventive and promotive services such as immunization; family planning; prenatal and postnatal care; birth preparedness and complication readiness; maternal, infant, and young child nutrition; personal hygiene; and home management and environmental sanitation. By 2014, though the implementation in developing regions and urban areas is in its infancy, a total of 2 289 741 1to5NLs had been formed in the country [14]. Recognizing the critical contribution of the HDAs in improving the health status of Ethiopians and to ensure the continuity and sustainability of health programs through community engagement, the government of Ethiopia set strategic objectives of which expansion of 1to5NLs being one of the focus areas for the coming years till 2020 [14].

## Methods

### Study setting

This study was conducted in Oromia and Tigray regions of Ethiopia. Oromia region is the largest state in Ethiopia while Tigray is the northernmost of the nine regions of the country. Both Oromia and Tigray regions are agrarian and the population size is projected at 35 467 001 and 5 247 005 in 2017, respectively [17]. According to the 2016 demographic and health survey, the total fertility rate was 5.4 for Oromia and 4.7 for Tigray. Infant mortality rate was 60 and 43 for Oromia and Tigray regions, respectively [18].The number of HEWs trained and deployed were 13 856 in Oromia and 1433 in Tigray until 2009 [19].

### Conceptual framework

**Fig. 1:**
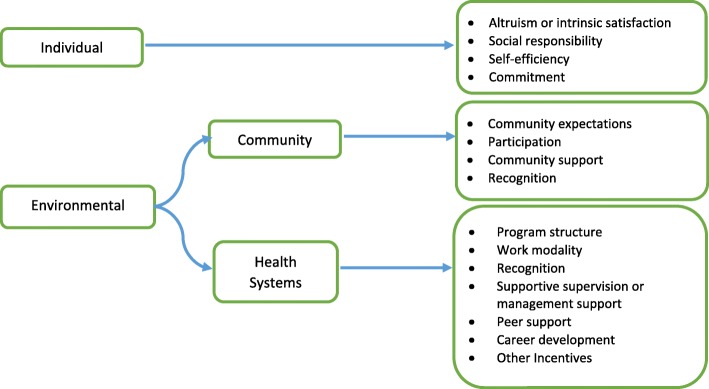
Conceptual framework of possible factors influencing community health workers’ motivation

Motivation of CHWs is affected by a complex set of factors. In this study, motivation is defined as an individual’s degree of willingness to exert and maintain an effort towards program goals [20]. We used a modified framework adapted from previous studies [13, 20, 21] to understand what actually motivate and demotivate CHWs. The sources of motivation can be internal to the health workers or they can be attributed to factors in their work and social environment [20]. Thus, the motivational factors in this study were broadly classified into individual and environmental factors where the latter was further divided into health systems and community-level factors (Fig. 1). We have also explored possible motivators and de-motivators of vCHWs in each of the above broad categories reviewing various studies conducted in developing countries [13, 21, 22].

The factors influencing motivation at individual level were intrinsic satisfaction, social responsibility, self-efficiency, and commitment [7, 13, 21]. Community expectation, recognition, participation, and support were the motivational factors recognized at the community level [13, 21, 23]. At the health system level, the structure of the program, work modality, recognition, supportive supervision, peer support, and career development were identified as the motivating and demotivating factors to CHWs [7, 9, 13, 21, 23].

### Measurement instrument, pilot testing, and training of data collectors

This cross-sectional study was conducted in September 2015 in four woredas of Tigray and Oromia regions, two woredas from each. The study used a quantitative, in-person survey tool, conducted with 1to5NLs currently working as volunteers in the selected woredas.

The data collection was part of a baseline survey to more broadly assess the knowledge, skills, and factors influencing motivational levels of the 1to5NLs. The initial motivational level set of questions included 34 factors expected to be associated with or predictive of motivation or demotivation. Motivational items were adopted from a study conducted in Iran by *Daneshkohan* et al. [9] and literature was reviewed on studies of the motivation of health workers to identify questions relevant to the Ethiopian context based on the reviewed literature and what actually the 1to5NLs are currently engaged in [7, 11, 12, 14, 22].Questions related to remuneration, timelines, and attendance were left out since the 1to5NLs are non-salaried and they have no fixed time to do their job and question on clearness of the HDA guideline was added. The questions were a 5-point Likert scale questions ranging from “strongly disagree” to “strongly agree”. Please see Table 2 in the “Results” section below for a list of the final constructs and factors used.

The data collection tool was pilot-tested in two non-study woredas in Oromia and Tigray regions on a total of 15 willing participants to test the validity of the questions. Some revisions were made based on the findings from the pilot test and the tool was finalized and ready for the training of interviewers or data collectors.

A 6-day training was provided to the survey team in each region. The training was intended to provide the survey personnel with a common understanding of the terms and definitions used in the survey tool and was highly participatory, with a mix of presentations, small group tasks, role-play, and field practice. The data collectors were also trained on techniques of interviewing and how to obtain appropriate consent from respondents. Further modifications of the data collection tool were also made during the training. Based on the pilot result and the training of interviewers, six constructs and 20 questions were finally developed for the actual data collection with regard to motivation.

### Sampling strategy and data collection

The study utilized a multistage cluster sampling framework to select 1to5NLs for the study. Two regions, Oromia and Tigray, were selected conveniently based on a recommendation from Ethiopia’s Federal Ministry of Health (EFMOH) for their better performance on HDA, commitment of woreda health officials to implement a pilot intervention after the assessment of the HDA, and proximity of the woredas to the regional health bureaus.

In each of the regions, two woredas were randomly selected. From each of the woredas, one of the approximately five health centers in the woreda was randomly selected. The catchment area of each health center covers approximately five kebeles, and from each kebele in the selected health center catchment areas, one ketena with an estimated population size of 2000 and comprising of three or more gotes (villages) was randomly selected. Finally, all the 1to5NLs in each ketena were selected and participated in the study.

All data were collected independently by trained survey team members in the two regions during the same 3-week period with close follow-up by the lead author. Survey teams in each region comprised of one regional coordinator, two supervisors, and 20 data collectors. The data were collected using hand-held devices or tablets on a template designed by Open Data Kit (ODK).^2^

### Data management and analysis

The data were exported from ODK to spreadsheet format, CSV file. Data cleaning, consistency checking, and verification of missing data were carried out using STATA 11 and the analysis was done using SPSS 20. Each Likert scale response was entered as a score of 1 to 5 where a score of 5 represents “strongly agree” and a score of 1 represents “strongly disagree” for positively worded questions and a score of 1 represents “strongly agree” and a score of 5 represents “strongly disagree” for negatively worded questions. A score of 3 in both positively and negatively worded questions represents “neither agree nor disagree”. Negatively worded questions were reversed so that higher percentages and mean scores indicate higher motivation. The reliability of the factors in measuring motivation was tested by Cronbach alpha. Despite its importance to see the overall effect of each factor in motivating and demotivating 1to5NLs, the difference in mean score among the factors was not that significant to concentrate the discussion in our study. Therefore, percentage of participants who agree and strongly agree to each of the motivational items was used to identify important motivating and demotivating factors. Comparison between attributes of social and work-related determinants in terms of selected demotivating factors was conducted using Mann-Whitney *U* test.

## Results

A total of 786 informants were approached and interviewed. Out of the interviewed informants, there were three (0.38%) incomplete questionnaires and these were subsequently dropped from further analysis.

### Background characteristics of the 1to5NLs

The average age of the respondents was 36 years with a minimum of 17 years and a maximum of 71 years (Table 1). Seventy-six percent of the interviewed respondents were aged 40 and less. The majority of the women (72.8%) were married during the survey time and about 96% of them have been married at least once in their lives. Approximately 65% of them had no formal education and from those who attended formal education, a larger proportion (88.2%) had completed at least some of primary school (grade 1–8) education. Almost 72% of the study respondents had worked as a 1to5NL for two or more years at the time of the survey. Twenty three percent of the respondents had prior volunteer experience in various health or development activities.

**Table 1:** Background characteristics of the study population

Characteristics	Number	Percentage (%)
Age (*n* = 745)		
30 or less	254	34.09
31–40	313	42.01
> 40	178	23.89
Mean (SD)	35.83 (8.94)	–
Minimum	17	–
Maximum	71	–
Marital status (*n* = 783)		
Single	34	4.34
Married	570	72.80
Divorced	102	13.03
Separated	12	1.53
Widowed	65	8.30
Formal school (*n* = 783)		
Yes	272	34.74
No	511	65.26
Highest level of education (*n* = 272)		
Primary	240	88.24
Secondary	32	11.76
Service year as a 1to5NL (*n* = 783)		
< 2 years	221	28.22
2–4 years	562	71.78
Prior volunteer experience in health or other development activities (*n* = 783)		
Yes	179	22.86
No	604	77.14

### Factors affecting motivational level of 1to5NLs

The list of factors that may affect motivation of 1to5NLs as measured by percentage of participants who agreed and strongly agreed for each of the factors and mean score is presented in Table 2. Internal consistency of the scale, as measured by Cronbach’s alpha, was found adequate (0.90).

**Table 2:** Factors influencing motivation of 1to5NLs

Construct	Factors	% and no. of participants agreed and strongly agreed	Mean score* (SD)
Job satisfaction	1- I am not satisfied with the work relationship I have with other 1to5NLsin my neighborhood	64.11 (502)	3.49* (1.13)
	2- I have a good working relations with the HEWs	75.48 (591)	3.71 (1.04)
	3- My effort is recognized and I get appreciation for my accomplishment from the HEWs and Kebele leaders	63.22 (495)	3.43 (1.09)
	4- I am happy with the status I have in the community as I volunteered for the HDA task	81.86 (641)	3.87 (0.95)
	5- I feel frustrated whenever I think I could not develop my career	51.47 (403)	3.27* (1.15)
Intrinsic job satisfaction	6- I am satisfied with the opportunity to use my abilities in my job	80.97 (634)	3.85 (0.94)
	7- I am satisfied that I accomplish something worthwhile in this job for the community	81.61 (639)	3.85 (0.94)
	8- I do not think that my work in the community as HDA is valuable these days	69.35 (543)	3.67* (1.08)
Commitment	9- I am glad that I volunteered to serve the community rather than engaging myself in other business	81.61 (639)	3.91 (0.94)
	10- I feel very little commitment to the community these days	72.92 (571)	3.71* (1.03)
	11- The community really inspires me to do my very best on the job	72.54 (568)	3.68 (0.97)
Conscientiousness	12- I always complete my tasks efficiently and correctly	77.91 (610)	3.82 (0.94)
	13- I am a hard worker	71.39 (559)	3.75 (0.98)
	14- I do things that need to be done without being asked or told	67.43 (528)	3.61 (1.00)
Management support	15- There is fair treatment by the Health Post (HP)/HEWs among 1to5NLs	76.76 (601)	3.76 (1.05)
	16- I get support from the HP/HEWs when problems arise	74.71 (585)	3.65 (1.08)
	17- The HP/HEWs really cares about my well-being	70.24 (550)	3.63 (1.07)
	18- The HP/HEWs shows very little concern for me in terms of frequent supervision and support	62.32 (488)	3.47* (1.12)
HDA structure	19- The guidelines is not that clearly stated	59.26 (464)	3.32* (1.18)
	20- There is lack of job description	63.98 (501)^1^	3.40* (1.17)

*Negatively worded questions were scaled as 1 (strongly agree) and 5 (strongly disagree). Therefore, a high mean score shows disagreement and thus suggestive of supporting higher motivation

As shown in Table 2, the highest percentages were observed for factors 4, 7, and 9, indicating that the majority of 1to5NLs strongly agreed that they are motivated more by having a good status in the community as a result of their volunteer activities (81.86%), serving the community rather than engaging in other businesses (81.61%) and the satisfaction that they get as a result of accomplishing something worthwhile to the community (81.61%).

The lowest percentage (51.47%) was observed for factor 5, suggesting that almost half of the 1to5NLs are more demotivated or frustrated whenever they think they cannot develop their career. Factors pertaining to the HDA structure, particularly, unclear guideline, also had the least effect in motivating 1to5NLs (59.26%). Moreover, limited supervision and support from immediate supervisors (62.32%) and lack of recognition and/or appreciation from supervisors and kebele leaders (63.22%) were also demotivating factors highlighted by the participants.

### Significance of selected demotivating factors among 1to5NLs

In order to assess how social status such as education and marital status, and previous volunteer experience and length of service affect motivation, three motivational questions were selected as they are found to be the major demotivating factors for the 1to5NLs as shown in Table 2. The significance of these determinants on the selected demotivating factors is summarized in Table 3.

**Table 3:** Significance of education, marital status, years of service, and previous volunteer service engagement on selected demotivating factors

Grouping variable		I feel frustrated whenever I think I could not develop my career		The HP/HEW shows very little concern for me in terms of frequent supervision and support		My effort is recognized and I get appreciation for my accomplishment from the HEWs and kebele leaders	
		Mean rank*	*P* value	Mean rank	*P* value	Mean rank	*P* value
Education	Formal	402.31	0.331	413.57	0.038**	429.09	0.000**
	No formal	386.51		380.52		372.26	
Marital status	Living in union	386.24	0.223	388.22	0.415	381.39	0.019**
	Not living in union***	407.41		402.12		420.39	
Service year as 1to5NL	< 2 years	422.89	0.012**	394.50	0.836	353.72	0.001**
	2–4 years	379.85		391.02		407.05	
Previous volunteer service	Yes	379.51	0.379	401.31	0.504	458.40	0.000**
	No	395.70		389.24		372.32	

*Mean of the ranks of each of the values of the determinants, at http://tqmp.org/RegularArticles/vol04-1/p013/p013.pdf. Accessed 11 July 2016

**Significant difference (*P* value < 0.05)

***Single, divorced, separated, widowed

The most frequent demotivating factor, “I feel frustrated whenever I think I couldn’t develop my career” was not significantly influenced by educational level, marital status, and previous engagement in volunteer service, *p* = 0.331, *p* = 0.223, and *p* = 0.379, respectively. However, service year as a 1to5NL affected the demotivating factor significantly, *p* = 0.012. The mean rank for service year shows that those 1to5NLs who had completed less than 2 years of service are more likely to be frustrated with thoughts of future career development than those with more than two service years. With the exception of educational level (*p = 0.038*), the other determinants had no significant effect on the perception of the 1to5NLs towards the HEWs’ concern for them in terms of frequent supervision and support. 1to5NLs with formal education are more likely to believe that they were not receiving the deserved supervision and support from HEWs than those with no formal education. The motivational level of 1to5NLs with respect to recognition and/or appreciation of accomplishments was significantly affected by education level (*p* = .000), marital status (*p* = .019), years of service (*p* = 0.001), and previous volunteer engagement (*p = 0.000*). 1to5NLs who had formal education, who were not living with life partners, who served for more years, and who had previous volunteer experience believed that their effort was recognized more and got appreciation from their supervisors and kebele leaders than their counterparts.

## Discussion

### Individual, community, and health system factors

The results of this study showed the degree to which a range of factors, individual, community, and health system factors, contributed to the motivation of vCHWs in Ethiopia. Health system factors were the most important deterrents of CHWs of which lack of career development was the primary discouraging factor. Consistent with this finding, a study in developing countries revealed that poor career paths and promotion opportunities lead health workers feeling stuck and demotivated [24]. An astonishing relationship was also observed between volunteering and career motives in a study conducted in South Africa; *Akintola* argued that the long-term sustainability of using poor and unemployed members of the community as volunteers in home-based care can be ensured through the creation of career paths [25].

Unclear guideline was the second health system-level demotivating factor the participants highlighted. Evidence showed that a well-considered guideline and job description should help to avoid any misconceptions on what volunteers do and could lead to better management [26]. Similarly, in a study conducted to assess the factors affecting the performance of community health workers in India, absence of clear guidelines and job descriptions among community health workers lead to greater noncompliance and poor performance [27]. *Kiangura and Nyambegera* reported that about 90% of volunteers in their study agreed that clear job expectation and methods of evaluation would motivate them to perform better [28].

Our study also showed that lack of frequent supervision and support from supervisors was another system-level demotivating factor. Evidence from different countries underlined the importance of supervision as a tool to motivate CHWs and reduce attrition. CHWs in Tanzania and Uganda mentioned that they were appreciative of the supervision made by their supervisors and that it increased their credibility and recognition in the community and found it to be highly motivating [29, 30]. Consistently, participants of a study in Mozambique and Madagascar pointed out the negative effects of irregular supervision on their performance [31, 32]. On the other hand, regular supportive supervision to CHWs was cited as a prerequisite for good quality care [3, 13, 33, 34].

Another health system-level factor, lack of recognition and/or appreciation of accomplishments, was affirmed by participants of our study as a demotivating factor. Evidences showed that appreciation or recognition of good performance by employers and communities is one of the incentives motivating health workers [35–39]. Consistently, WHO stated that financial incentives are not the only factors that cause lack of motivation but also non-financial incentives such as appreciation and recognition [40].

On the other hand, this study showed that community-level factor, having a good status in the community, was the first factor motivating the 1to5NLs. Evidences showed that community acceptance affects performance and motivation of CHWs and is a significant predictor of retention [23, 41]. Being identified as a CHW and affiliated with the health system is usually, though not always, considered as having a status that generates power and respect within a community [42]. When CHWs in Nepal were asked why they continued their volunteer service, they said, “Our neighbors won’t let us resign; they insist we continue because their children’s health depends on us” [43]. Public honoring, involvement in public meetings, more participation in community decision meetings, and more invitations in various events are some of the means to improve social prestige of CHWs [13, 44]. vCHWs in Ethiopia said that an event organized to thank them in front of the community would strengthen their motivation [22]. In Indonesia, a radio-based health communication campaign motivated the CHWs by publicly praising them as “volunteers who work without compensation for our children in our village for the sake of the future” [45]. However, adverse sentiments from the community are a potential reason for poor retention of CHWs [41].

Individual-level factors were also repeatedly mentioned by participants of our study as motivating factors. Commitment to serve the community as 1to5NLs rather than engaging in other businesses and accomplishing something worthwhile to the community were the second set of motivating factors of the respondents. Such compassion and commitment to serve the community may emanate from having acquainted with the existing health condition of their society. In Tanzania, CHWs were attracted to public services and find personal satisfaction and pride in helping their communities, as expressed by the desire to provide education and prevent common tragedies like the loss of a child [7]. Similarly, a quantitative study on vCHWs in northwestern Tanzania found that more than three quarters of the CHWs continue to volunteer as they enjoy serving the community [46]. According to a WHO report, health workers appear to be strongly motivated by observed reductions in burden of disease over time, and their own internal sense of purposefulness and honor about a job well done [42, 47].

In general, participants of this study are motivated by individual- and community-level factors while they are more likely to be demotivated by system-level factors. As it is very difficult to sustain volunteers’ interest strictly with remuneration in low-income countries [25], other ways of addressing motivation of vCHWs should be considered. Notwithstanding the intrinsic sources of motivation and community acceptance, long-term sustainability of volunteer CHW programs usually require supportive and responsive health system [48] one of which is the creation of a platform that allows unemployed volunteers to fulfill their motives through skill training, thereby improving their employment opportunities in the labor market [25]. Thus, given that 1to5NLs are not stipend, ensuring their satisfaction and consequently improving their motivation should be given due attention by the health system so that they can continue providing volunteer services.

### Significance of selected demotivating factors

Despite insufficient evidence to draw conclusion on how each 1to5NL valued motivational factors, our study examined the effect of educational level, marital status, previous engagement on volunteer activities, and years of service on three of the identified de-motivational factors: lack of career path, lack of recognition and/or appreciation of accomplishments, and infrequent supervision and support. The current study found that the first demotivating factor, “I feel frustrated whenever I think I couldn’t develop my career”, was not significantly affected by educational level, marital status, and previous engagement in volunteer service. However, 1to5NLs with less than 2 years of service year felt the lack of career path more than those with more service years; the larger proportion (42%) in the former group were aged 30 years or less and another 41% between 30 to 40 years. Given the very high prevalence of youth unemployment in Ethiopia and globally [49] and younger people tend to be literate [42], the difference in the perception of lack of career path may be attributed to the desire to develop one’s career and to be employed in money-earning jobs is more pronounced by younger individuals than older ones.

Education was the only factor that had a significant effect on the perception of 1to5NLs about how frequently they received supervision and support. Comparing with volunteers with no formal education, network leaders who had a formal education are more likely to report dissatisfaction with the amount of supervision and support that they received when compared to those who had not received any formal education. HEWs are usually occupied with various tasks at the health post, house-to-house visit, collecting supplies from health centers, mobilizing the community for developmental activities, and training and supporting 1to5NLs. Moreover, there may be an average of 100+ 1to5NLs under each HEW. Thus, allocating supervision time equally among the 1to5NLs might have been be a challenge for the HEWs and perhaps put much of the time on those with no or low education.

Recognition and/or appreciation of accomplishments has previously been cited as one of the most important motivating factors for CHWs [36, 39]. In our study, it was significantly affected by education, marital status, length of service, and previous volunteer service engagement. 1to5NLs who had no formal education, who were living in union, who served for less years, and had no previous volunteer engagement were more likely to report dissatisfaction with the amount of recognition and appreciation that they received from their supervisors than their counterparts. The dissatisfaction of the 1to5NLs with no formal education on recognition and appreciation of accomplishments may stem from the desire to get more attention and consequently more support as they are non-literate and use only illustrations to educate their group members and record information. Despite the burden on married CHWs in rural areas due to child rearing and domestic chores, family attitude towards their role as CHW and whether they experienced family disapproval may encourage or discourage the CHWs to continue as volunteers. Studies have shown that familial disapproval was found to be a barrier for volunteer tasks and a reason for resigning particularly for women whose husbands saw the long hours laid on such activities inappropriate [50–52]. Thus, volunteering for the job, executing their responsibility as a HDA network leader coupled with other competing tasks at home, and the challenge of possible families’ or husbands’ disapproval might have contributed to the higher expectation of married 1to5NLs who are living with their partners to be recognized and appreciated by their supervisors than their counterparts.

Consistent with our study, while more experienced volunteers who served for many years in previous or current position were settled or integrated well within their community, the newly recruited CHWs usually faced challenges and require more recognition and support from their supervisors [33, 53, 54]. Mutale et al. in their study had observed that the longer the health workers stayed in post the more motivated they were [54]. Similarly, a study in a Kenyan district found that CHWs who had served for more than 3 years were twice more likely to mention that they are being motivated to serve their community than those who had served for less than 3 years [55].

### Limitations of the study

We employed only a quantitative method based on questions tested in other contexts. Due to time and other resource constraints, the study did not include a qualitative approach that would have helped to explore how and what conditions would motivate the 1to5NLs. In addition, the study was based on questions asked directly to the 1to5NLs and did not assess their performance. Although the questions were worded both positively and negatively to reduce response bias, the answers might have been influenced by the respondents’ perception to each of the question and what they think the investigators wishes to hear/social desirability bias. Looking further to the data, we have also observed that the percentage of “Score 1” to each of the motivational items is very small, suggesting the existence of central-tendency bias in their response (a condition where respondents are hesitant to rate attributes at the extremes of the scale and prone to rate most attributes at the center of the scale; one of the weaknesses of Likert scale). As a result, the mean score to each of the motivational factors fall between 3 and 4 on the scale. Another weakness of the Likert scale is that the space between each scale cannot possibly be equidistant. Thus, the result of this study should be interpreted keeping in mind that degree of literacy level of the respondents to comprehend the questions, difficulty to distinguish the scales or response categories, insufficient attention to questions due to time pressure, lack of fluency of interviewers in asking the questions, and other cultural reasons might have affected the responses of the study participants. Furthermore, the result may not represent all 1to5NLs in the country as the factors contributing to their motivation level may vary from region to region depending on the knowledge and skills of their supervisors and the support they get from kebele councils.

## Conclusions

The findings of this study have shown that individual, community, and health system factors have contributed to the motivation of 1to5NLs. Social acceptance, enthusiasm to serve the community, and accomplishing something worthwhile to the community were the factors motivating the network leaders to engage and stay in volunteer services. In this regard, the health system needs to create a platform where the community actively involves in harvesting its own health and make sure that the HDAs are empowered by promoting the work that they do is worthwhile using regular meeting and media such as radio-based communication to improve their motivation and retention and possibly attract more volunteers to the business.

In addition, the study identified possible factors negatively affecting the motivational levels of 1to5NLs of which lack of career development being repeatedly reported by the respondents. In situations where resources are constrained and volunteers are not receiving stipends, alternative incentive mechanisms should be considered. We, therefore, recommend that the EFMOH rethink current policies and plan to create career development models to achieve a more positive impact in the development of primary health care by motivating 1to5NLs and attracting more youth volunteers to community health services.

As underlined by 1to5NLs, infrequent supervision and inadequate support from immediate supervisors and lack of recognition and/or appreciation for accomplishments were other factors dissatisfying the study participants. Therefore, we recommend that strategies should be developed to strengthen frequent and regular collaborative supportive supervision by the HEWs and supervisors from health centers and woreda health offices to show government’s commitment in sustaining the program and thereby encouraging and motivating the 1to5NLs to do their job with passion. Additionally, beyond the need to recognize and appreciate efforts of 1to5NLs by immediate supervisors during their meetings, we recommend the use of non-financial incentives, such as trophies, certificates, and other tokens of recognition and appreciation by kebele and woreda administration in front of the public. Finally, the study has shown the need to conduct more rigorous researches to make appropriate clarification on the guideline of HDA structure to overcome any misapprehensions on what 1to5NLs do.

## Abbreviations

1to5NLs: One-to-five network leaders
CHWs: Community health workers
EFMOH: Ethiopia’s Federal Ministry of Health
HDA: Health Development Army
HEWs: Health extension workers
HP: Health Post
JSI: John Snow Inc.
ODK: Open Data Kit
SD: Standard deviation
vCHWs: Volunteer community health workers
WHO: World Health Organization

## Footnotes

1 The lowest administrative structure in rural Ethiopia

2 Free and open-source set of tools to manage mobile data collection. http://opendatakit.org accessed. Accessed 27 July 2015
